# Disinformation and Democracy on the Docket: Reformulating the Approach to Electoral Disinformation under the ECHR

**DOI:** 10.1093/ojls/gqaf026

**Published:** 2025-07-23

**Authors:** Katie Pentney, Ethan Shattock

**Keywords:** democratic rights, free elections, freedom of expression, disinformation, foreign information manipulation and interference, European Convention on Human Rights

## Abstract

With the pending case of *Bradshaw and others v United Kingdom*, the European Court of Human Rights finds itself at a crossroads: it can either cement its free elections jurisprudence under article 3 of Protocol 1 (P1-3) of the European Convention on Human Rights or it can recalibrate and refine it to better safeguard the electorate’s democratic rights in the face of electoral disinformation and foreign information manipulation and interference. This article makes the doctrinal and normative case for the latter option. We scrutinise three limitations in the jurisprudence: first, the Court’s individualised approach to electoral falsehoods under P1-3, at the expense of the *electorate’s* rights as informed democratic participants; second, the focus on *reactive* positive obligations to combat electoral disinformation, rather than proactive measures to ensure the free expression of voter choice; and finally, the lack of clarity about how the rights to free elections and to freedom of expression should be read harmoniously where they conflict.

## 1. Introduction

The use of disinformation to distort democratic elections is at the forefront of European scholarly, policy and legal debates.^1^ Election interference and the manipulation of information for political purposes are not new phenomena;^2^ however, digital technologies have emboldened actors spreading false and misleading information to distort voter choices in democratic electoral processes at home and abroad.^3^ In response, legislation designed to curtail the spread and mitigate the effects of disinformation and foreign information manipulation and interference (FIMI) in electoral processes has proliferated in recent years.^4^ Scholarly critiques abound on the potential rights infringements such anti-disinformation efforts pose, in particular to freedom of expression and to free and fair elections.^5^ Less academic inquiry has been devoted to the inverse question: that is, whether a state’s *failure* to address disinformation may undermine these same rights.^6^ This article offers a doctrinal and normative response to this question, through the prism of the right to free elections recognised in article 3 of Protocol 1 (P1-3) of the European Convention on Human Rights (ECHR, the Convention). P1-3 provides:

The High Contracting Parties undertake to hold free elections at reasonable intervals by secret ballot, under conditions which will ensure the free expression of the opinion of the people in the choice of the legislature.^7^

Elucidating states’ obligations in respect of electoral disinformation and FIMI is not only an important contribution to ongoing scholarly, policy and judicial debates, but an increasingly urgent imperative. Romania recently became the first country in the European Union to cancel an election over FIMI, following reports of Russian disinformation on TikTok.^8^ In annulling the first round vote, the Constitutional Court of Romania highlighted the state’s obligation to safeguard electoral integrity, which has two components: first, the state’s positive obligation ‘to prevent any unjustified interference in the electoral process’; and second, ‘a duty of neutrality, which includes the obligation to strengthen the resilience of voters’.^9^ In sum, the Constitutional Court held that ‘the state must address the challenges and risks posed by organised disinformation campaigns that may affect the integrity of electoral processes’.^10^ By contrast, several years earlier, courts in the UK reached a markedly different conclusion in a challenge brought by Members of Parliament (MPs) and others to the government’s failures to implement an effective regulatory framework to address Russia’s disinformation and FIMI during the 2017 general election, the 2016 Brexit referendum and the 2014 Scottish independence referendum.^11^ The High Court refused the request, holding, in part, that it was not arguable that specific legal obligations—which endeavour to prescribe the detailed structure or shape of electoral law—existed under P1-3 of the Convention.^12^

In the face of seemingly contradictory domestic judgments, the European Court of Human Rights (ECtHR, the Court) will soon weigh in on whether state failures to address disinformation may undermine the right to free elections. The case of *Bradshaw and Others v United Kingdom* was communicated by the Court in December 2022.^13^ The applicant MPs alleged that the UK government failed to protect its institutional obligation to ‘hold free elections’ as required under P1-3.^14^ The application to Strasbourg arose from two independent reports into Russian disinformation operations in UK elections. In February 2019, the Digital, Culture, Media and Sport Committee released its report on disinformation and ‘fake news’, in which it called on the UK government to launch an independent investigation into the nature and extent of foreign influence and disinformation campaigns during the 2017 general election, the 2016 Brexit referendum and the 2014 Scottish independence referendum.^15^ In July 2020, the Intelligence and Security Committee of Parliament (ISC) released its final report on its inquiry into Russian interference.^16^ The ISC indicated that ‘the clearest requirement for immediate action is for new legislation: the Intelligence Community must be given the tools it needs and be put in the best possible position if it is to tackle this very capable adversary’.^17^ The ISC further stated that protecting democratic discourse and electoral processes against foreign interference ‘is a central responsibility of Government, and should be a ministerial priority.’^18^

Despite these reports, the UK government declined to launch an independent investigation into Russian interference in the country’s democratic processes. Accordingly, three applicant MPs—together with two life peers and a non-profit organisation—sought permission to challenge, by way of judicial review, the UK government’s failure to do so, as well as its failure to implement an effective regulatory framework to ensure the conditions for the free expression of the opinion of the people. Both failures were alleged to violate P1-3.^19^ The High Court refused to grant permission, finding *inter alia* that P1-3 did not compel the government to launch an independent investigation.^20^ A further application for permission to appeal on ECHR grounds only was refused on similar grounds: the High Court judge found that no specific legal obligations prescribing the detailed structure or shape of electoral law existed under P1-3.^21^ The Court of Appeal refused the application for permission to appeal. The applicants thus took the matter to Strasbourg.^22^

This article posits that *Bradshaw* could be a seminal judgment in recognising election disinformation and FIMI as potential rights violations.^23^ Such recognition would have repercussions throughout the Council of Europe (CoE), particularly in light of the recent Romanian elections and ongoing efforts across Europe to curb electoral disinformation and FIMI. Perhaps more importantly, however, *Bradshaw* affords the Court the opportunity to recalibrate and refine the P1-3 jurisprudence to better safeguard the electorate’s democratic rights. As the ensuing discussion reveals, such opportunities do not come around very often, and should be seized. It may be some years yet before the application is decided,^24^ and the judgment will undoubtedly spur scholarly critique and consideration. At this liminal moment, we contend that the Court is at a crossroads: with *Bradshaw*, it can either cement its existing jurisprudence, with all its shortcomings, or it can break new ground in protecting Convention rights in the face of electoral disinformation and FIMI. This article makes the doctrinal and normative case for the latter option. In doing so, it seeks to proactively guide the Court in this endeavour and to provoke scholarly debate about states’ positive obligations in the face of FIMI and electoral disinformation threats.

This article identifies and critiques three conceptual limitations in the existing ECtHR jurisprudence which the Court can—and should—address in *Bradshaw*. First, the Court should move beyond its individualised approach to P1-3 in cases involving electoral falsehoods. To date, the Court has largely focused on the scope and nature of *individual* candidates’ rights to stand for election, with only marginal focus on how the dissemination of false and/or misleading information can undermine the rights of the *electorate* as informed democratic participants. Second, the Court should recognise and develop states’ *proactive* positive obligations to combat electoral disinformation, moving beyond its traditional reliance on reactive measures. Procedural investigations after rights infringements have occurred and the damage caused by electoral falsehoods has been done are necessary but not sufficient. The Court should promote meaningful upstream protection for electoral processes while providing guidance to CoE states about the measures necessary to ensure the free expression of voters’ choices in the choice of the legislature. Finally, the Court should clarify the interaction between the rights to free elections under P1-3 and to freedom of expression under article 10 ECHR. While the Court has recognised that these rights are ‘inter-related and operate to reinforce each other’, uncertainty remains about how states should mediate these rights to protect the electorate from being deceived during elections.^25^  *Bradshaw* provides an opportunity for the Court to flesh out how states can address the interference occasioned by electoral disinformation and FIMI while ensuring compatibility with relevant Convention provisions.

In what follows, we map the relevant ECtHR jurisprudence; critique its limitations for meaningful protections of the electorate’s rights; and explain how the Court can and should address such limitations in *Bradshaw*. In light of the rapid proliferation of regional and domestic laws in Europe designed to address election falsehoods—and recent domestic judgments annulling electoral results on the grounds of disinformation and FIMI by Russian actors—the Court’s interpretive guidance in this area is vital.^26^ This article provides doctrinal and normative building blocks to move the Court beyond its existing posture to better protect the democratic values upon which the Convention was founded and the democratic and expressive rights enshrined therein.

## 2. Shifting from the Individual to the Electorate

This section sets out and critiques the first limitation in the P1-3 jurisprudence: namely, that it evinces a focus on individual rights, to the detriment or exclusion of the electorate’s rights.^27^ While the right to free elections expressly imposes a positive duty on states to hold free elections, the Court has gradually inferred two individual rights from the institutional obligation to hold elections under P1-3: the right to vote (the ‘active aspect’ of P1-3) and the right to stand for election (the ‘passive aspect’ of P1-3).^28^ The identification of individual electoral rights provides greater texture to P1-3, yet the Court’s focus on these individuated dimensions of P1-3 has masked the provision’s broader purpose: namely, safeguarding the interests of the electorate.^29^ The jurisprudence on the requisite conditions to ensure the free expression of the electorate—what could be called the ‘public aspect’ of P1-3—remains stunted and underdeveloped.

Two strands of the P1-3 jurisprudence illustrate this individual focus: first, cases concerning sanctions imposed on electoral candidates for purveying false information to electoral commissions; and second, cases concerning alleged vote tampering and irregularities which could affect election results. Both strands cast the rights inquiry narrowly, probing the impacts on individual candidates’ rights rather than on the electorate’s right to a free election. This individualised framing stems from the factual underpinnings in most P1-3 cases and the stringent standing requirements for victims of rights infringements to bring an application to Strasbourg. The *Bradshaw* application differs in important respects from these precedents; it may thus enable the Court to move beyond this individuated prism to assess the broader interest of maintaining an informed electorate.

We begin by distilling the relevant jurisprudence, before explaining the limitations of the Court’s individualised approach and, finally, proposing the potential course correction afforded by *Bradshaw* to better protect the electorate’s democratic rights.

### A. The Blame Game: Culpability for False and/or Misleading Electoral Information

Where electoral candidates have been sanctioned for providing false or misleading information to electoral commissions, the judgments in respect of P1-3 typically turn on whether the applicant: (i) did so knowingly or intentionally; or (ii) had good reasons for doing so. An example of the former case is *Sarukhanyan v Armenia*.^30^ The applicant had provided false information about his assets and was accordingly deregistered as a candidate in parliamentary elections.^31^ The ECtHR accepted that—in principle—deregistration served an ‘undoubtedly legitimate’ aim of preventing voters from being ‘misled by false representations’.^32^ However, the Court found a violation of P1-3 because the applicant had not acted in bad faith in misrepresenting his property status; to the contrary, he had ‘good reason to believe that the information was accurate’.^33^ Accordingly, his omission ‘could not reasonably be blamed on him’.^34^ Implicit from such language is the distinction drawn between erroneous and intentionally deceptive false declarations; the Court focused on the electoral candidate’s underlying intentions when communicating falsehoods.^35^ To the extent the Court considered the effects on the electorate, it stressed that the information itself—regarding technical details on the candidate’s property status—was of ‘minor importance’ to voters,^36^ and that the factual discrepancy was not ‘seriously capable of misleading the electorate’.^37^

The ECtHR’s concern for individual candidates’ knowledge of and culpability for false information is epitomised in *Krasnov and Skuratov v Russia*.^38^ The applicants were disqualified from standing in parliamentary elections for submitting false information to election authorities. In considering the applicants’ P1-3 claim, the Court reasoned that ‘The requirement to submit information on the candidate’s employment and party membership serves to enable voters to make an informed choice with regard to the candidate’s professional and political background’.^39^ Moreover, asking candidates to ensure the accuracy of the information submitted was found to be ‘incontestably legitimate … lest the voters be misled by false representations’.^40^ Turning to the proportionality of the applicants’ disqualification, the Court made a critical distinction between the first applicant and the second. The first applicant had claimed to be the head of a district council even though he had not held the position for several months.^41^ The Court found he had knowingly provided ‘substantially untrue information’ and ‘cloaked himself in the authority associated in the voters’ eyes with a position he no longer held’.^42^ This factual discrepancy ‘was not a matter of indifference for the voters’, and the Court held that his falsification ‘could have adversely affected their ability to make an informed choice’.^43^ Accordingly, the Court reasoned that his provision of false information could have ‘adversely affected’ the electorate’s ‘ability to make an informed choice’.^44^

Conversely, no deceptive conduct was found in respect of the second applicant, who had listed his position as acting head of a law department while also employed as a professor in the department. The Court disagreed with the ‘inconsistent findings’ of the domestic authorities regarding the misleading nature of his submission.^45^ Differentiating this submission from the first applicant’s, the ECtHR found it crucial that the second applicant’s declaration did not suggest he had acted in bad faith.^46^ Moreover, the Court doubted that the second applicant’s discrepancy ‘was capable of misleading the voters’ in a manner that affected their vote.^47^ Thus, the Court found Russia’s interference with P1-3 to be justified in respect of the first applicant but not the second.^48^ The ECtHR appears more willing to accept states’ sanctioning of electoral candidates where they submit false information in an intentionally deceptive manner, and/or where the falsehoods are likely to influence voters’ choices.^49^

By contrast, the applicant candidate’s justification for supplying inaccurate information to an electoral commission was crucial in *Melnychenko v Ukraine*.^50^ Here, the applicant did not dispute that he had *knowingly* submitted inaccurate information concerning his ‘habitual’ residence.^51^ However, he argued that he had not intended to deceive voters: instead, the false declaration was attributable to his ‘fear of persecution in Ukraine’ and his desire to maintain his ‘personal safety or physical integrity’.^52^ The ECtHR held that Ukraine had violated the candidate’s right to stand for election as the applicant had a justifiable reason—not motivated by deceitful intent—for the false disclosure. This stance underscores how the Court analyses knowledge of falsity and intent to deceive in order to disentangle ‘the cut and thrust of political debate’ from ‘tactics’ that actively mislead citizens engaging with the electoral process.^53^

### B. Shifting the Dial: Vote Tampering and Electoral Irregularities

The ECtHR’s approach to P1-3 cases involving alleged election tampering and irregularities also has relevance in the context of electoral disinformation and FIMI. Where the Court has addressed these issues, it has focused on whether vote tampering or count irregularities could likely shift electoral outcomes. While this broadens the focus beyond the individual candidate’s culpability, the analysis still turns on the effect of the irregularities and tampering on the *candidate’s* prospects for victory. The electorate is considered only in respect of whether the restriction of the candidate’s rights thwarted the free expression of the electorate’s wishes.^54^

The Court’s inquiry in such cases has centred on whether an evident and decisive causal link exists between election irregularities and election outcomes. In *Babenko v Ukraine*, the applicant alleged that electoral ballots had been ‘mixed up’ and that voting irregularities had skewed the election result to his detriment.^55^ The Court dismissed the application because the applicant failed to demonstrate how the alleged irregularities had ‘specifically affected’ the election outcome.^56^ As the applicant had received 10,000 fewer votes than the opposition, the Court doubted that any alleged distortions had shifted the results in an electorally decisive manner.^57^

Similar reasoning prevailed in *Kerimova v Azerbaijan*, wherein two election officials confessed to having tampered with election protocols to disadvantage the applicant.^58^ Even so, the applicant received the most votes; nonetheless, the Central Election Commission invalidated the election results. The Court found that Azerbaijan’s invalidation violated P1-3 because of insufficient evidence that the irregularities in five polling stations had seriously affected election results.^59^ Notably, the Court reiterated that the goal of free elections is to identify ‘the opinion of the electorate’, but cautioned against ‘a situation where a winning candidate is wrongfully punished by being deprived of his or her victory in the election’.^60^ This conclusion may be contrasted with *Davydov and Others v Russia*, where the applicant electoral candidates complained that the electoral commissions had ‘falsified the results of the elections by ordering recounts’ that ‘systematically’ increased the ruling party’s share.^61^ Crucially, these allegations were corroborated by third-party election observers.^62^ The ECtHR found a violation of P1-3 because the investigations into alleged tampering had been limited to ‘trivial questions of formalities’ while ‘ignoring evidence pointing to serious and widespread breaches of procedure and transparency requirements’ that could plausibly have affected the election outcome.^63^

The Grand Chamber’s judgment in *Mugemangango v Belgium* also evinces a focus on the electoral outcomes of vote tampering for individual candidates.^64^ The applicant failed to win a seat in parliamentary elections by just 14 votes; he requested a recount as thousands of votes had been declared spoilt and may have been erroneously disqualified. The Grand Chamber found that the new parliament’s refusal to allow the recount violated P1-3.^65^ It was important that the applicant’s allegations were ‘sufficiently serious’, as he ‘might have been declared elected following the recount he was seeking’.^66^ Thus, by failing to address the applicant’s concerns, Belgium had not only interfered with the applicant’s right to stand for election, but had also failed to proactively investigate irregularities as required under P1-3.

The foregoing cases reveal the Court’s individualistic focus in cases of electoral falsehoods or tampering. The Court appears more inclined to accept states’ restrictions on the right to stand for elections if individuals have *knowingly and intentionally* purveyed false information which is likely to influence individual voter choices. Equally, the Court is more likely to find a violation where election tampering or irregularities may have seriously affected the election *outcome* to the candidate’s detriment. However, these cases were brought by electoral candidates and assessed in terms of how they were personally implicated by potential wrongdoings within the context of the impugned electoral event. This narrow and individualised focus on candidates’ rights masks the broader public interests and institutional processes P1-3 was enshrined to protect, as explained in more detail below.

### C. From Positive Rights to Individual Claims

The above cases illustrate a narrow focus on the P1-3 rights of the individuals concerned, with less attention paid to the rights of the electorate or the broader interest of maintaining an informed populace. The focus on ensuring the conditions necessary for free and fair elections is thus secondary, to the extent it is considered at all.^67^ This makes some logical sense: the Court must consider the alleged rights violation that is squarely before it. Each of the cases concerned an interference with the applicant’s ability to stand for election or secure their seat in the legislature. This granular interference prompts the application to Strasbourg and justifies the Court’s examination of the issues, and its explication of the scope and purposes of P1-3.

The Court has recognised that the right to free elections entails both an individual right and a positive obligation on the state, and covers a range of guarantees ‘starting from the right of the voters to form an opinion freely, and extending to careful regulation of the process in which the results of voting are ascertained, processed and recorded’.^68^ Yet these dual aspects of the right—as comprising an individual element and a broader obligation to the public—have not developed equally. This differentiated development has diluted the proclaimed essence of the rights guaranteed under P1-3 (which are ‘crucial to establishing and maintaining the foundations of an effective and meaningful democracy governed by the rule of law’^69^) and delayed its full realisation. In the context of electoral disinformation and FIMI, this ought to be cause for concern: the ECtHR has progressively fleshed out the scope and limitations of individual electoral rights under P1-3, while the development of states’ positive obligations under P1-3 to protect an informed populace have stagnated. The Court itself has acknowledged the difficulty inherent in achieving ‘a compromise between the requirements of defending democratic society on the one hand and protecting individual rights on the other’.^70^

Yet, beyond the passing reference to legitimate aims for CoE states to combat falsehoods and protect voters’ rights to make informed choices, the Court’s concern for the interests of the electorate has been comparatively muted. This is hard to square with the Court’s recognition that these rights serve to protect the public’s right to be informed,^71^ and that ‘free elections and freedom of expression, particularly freedom of political debate, together form the bedrock of any democratic system’.^72^ Freedom of expression generally, and within elections specifically, not only engages speakers’ rights, but also *audiences’* rights; the right ought to be concerned with the ‘protection of (and perhaps even support for) a relationship of communication’.^73^ The traditional understanding that ‘the key to a well-informed citizenry lay within *speech rights*’ has been questioned in the face of mounting evidence to the contrary.^74^ Rubén Marciel argues that a shift in focus is necessary, ‘from *speech rights* towards the *rights of the public*’ in which we ‘tak[e] more seriously the idea that citizens have a right to be well informed’.^75^

To date, the P1-3 jurisprudence has developed primarily around individual rights, specifically those of electoral candidates; to the extent P1-3 protects the electorate more generally, it is limited to ensuring that the conditions imposed on the candidate do not

thwart the free expression of the people in the choice of the legislature—in other words, they must reflect, not run counter to, the concern to maintain the integrity and effectiveness of an electoral procedure aimed at identifying the will of the people through universal suffrage.^76^

Something has been lost in translating the positive rights and public purposes of P1-3 into individual claims for relief before Strasbourg.^77^

### D. *The Potential of* Bradshaw

*Bradshaw* may make a significant contribution to the development of the P1-3 jurisprudence by reorienting P1-3 beyond a purely individualised lens to better protect the electorate’s rights. Put differently, while the active and passive aspects of P1-3 have been foregrounded to date, *Bradshaw* provides an opportunity for the Court to breathe life into the public aspect of the right. As explained, the applicants are MPs who have brought the claim in that capacity. However, unlike the above-discussed cases (or the recent application of the Romanian presidential candidate), they are not challenging a state’s action to sanction or bar them from personally participating in the electoral process. Nor are they challenging voting irregularities which impaired their electoral success. Instead, they are challenging the state’s failure to fulfil its positive obligation to the *electorate at large*: that is, its failure to ensure the conditions necessary for the free expression of the people’s opinion in democratic electoral processes. Such conditions were not met, they argue, because of FIMI—including the weaponisation of disinformation—during the independence referendums in respect of Scotland and the European Union and during a previous national election. Despite credible reports documenting Russian interference in British elections, the UK government failed to launch an independent investigation or to establish an effective regulatory framework to address such interference.

While the ECtHR’s recognition that P1-3 protects individual electoral rights is important, the protective scope of P1-3 should not be limited to individual rights at the expense of its broader purposes or the Convention’s democratic values. With *Bradshaw*, the Court can elucidate the scope and requirements of the (comparably underdeveloped) *public* aspect of P1-3, beyond the interpretive confines of personally affected electoral candidates seeking to vindicate their individual rights. A more holistic approach to states’ positive obligations to secure the free expression of the people would also be consistent with the inclusion of P1-3 and with its hard-fought textual construction.^78^ The exclusion of democratic rights from the Convention set it apart from the Universal Declaration of Human Rights (which recognised, in article 21, that ‘The will of the people shall be the basis of the authority of government; this will shall be expressed in periodic and genuine elections’).^79^ The exclusion of a right to free elections in the Convention was due, in part, to the antagonistic drafting process, wherein the reference to the ‘expression of the will of the people’ was met with significant opposition (in particular, by the UK delegation).^80^ However, states’ obligation to hold free elections ‘under conditions which will ensure the free expression of the opinion of the people in the choice of the legislature’ was ultimately included in the First Protocol, in recognition of the importance of a provision safeguarding the political rights of the citizenry within the Convention.^81^

Of course, the applicants in *Bradshaw* face two challenges in foregrounding the state’s alleged violation of the *public’s* democratic rights in the face of electoral disinformation and FIMI: the first concerns the victim status requirement, and the second concerns the margin of appreciation afforded the state. Article 34 ECHR requires that applicants must have been ‘the victim of a violation’ by the state, while article 35 requires that they suffered a ‘significant disadvantage’ in order for their claim to be admissible.^82^ The Court does not allow for complaints *in abstracto*, nor does the Convention recognise *actio popularis*, that is, applications brought in the public interest simply on the basis that a public action undertaken appears to be in breach of the Convention.^83^ Indeed, whether the applicants satisfy the victim status requirements—‘as sitting Members of Parliament’—is one of the questions the Court has put to the parties in *Bradshaw*.^84^ However, the Court’s approach to the victim status requirement is in a state of some flux, following the Grand Chamber’s judgment in *Verein KlimaSeniorinnen Schweiz and Others v Switzerland*.^85^ The Grand Chamber accepted that a Swiss association of elderly women had standing to challenge the state’s failures to combat climate change on behalf of its members; by contrast, the individual applicants did not have standing.^86^ The Court’s recognition of the representative standing of associations has been circumscribed to the climate change context, and would in any event not be directly applicable to the facts in *Bradshaw*.^87^ Whether the Court will find that the MPs in *Bradshaw* have standing as individual victims, or as representatives of their (specific or general) constituents, remains to be seen; it is clear, however, that its analysis on this point will have ramifications for the development of the electorate’s rights under P1-3.

The margin of appreciation afforded states may pose an additional hurdle. The margin of appreciation for states to manage elections is generally wide, accounting for the ‘numerous ways of organising and running electoral systems and a wealth of differences, *inter alia*, in historical development, cultural diversity and political thought within Europe’.^88^ Alain Zysset argues that this evinces the Court’s ‘timidity in electoral matters’, particularly where states are balancing competing or conflicting rights.^89^ The Court may be reluctant to set prescriptive requirements for states to fulfil their positive obligations to the electorate, particularly in light of the facts presented in *Bradshaw* and the nature of the applicants advancing the claim. However, as discussed in the next section, the Court has already set some minimum standards for states in P1-3 cases. Doing so in *Bradshaw* would be in line with its purposive interpretative approaches to Convention rights generally, and with its P1-3 jurisprudence specifically.^90^ Moreover, the Court has held that the margin of appreciation ‘is limited by the obligation to respect the fundamental principle of [P1-3], namely “the free expression of the opinion of the people in the choice of the legislature”’.^91^

The issues raised in the application engage states’ institutional obligations to safeguard democratic processes, and the Court’s explication of these obligations is relevant throughout the CoE. The pertinence and urgency of these issues are all the more evident in light of the recent annulment of the Romanian presidential election on the basis of FIMI and Russian disinformation in the pre-election period.^92^ Despite the challenges posed by restrictive standing requirements and the wide margin of appreciation afforded states in P1-3 cases, the Court may find that examination of the *Bradshaw* application on the merits is warranted, on the basis that respect for human rights as defined in the Convention requires it. The Court can, and should, take this opportunity to put ‘the people’ back into P1-3 jurisprudence; that is, it should place the electorate’s rights, and the broader interest of maintaining an informed electorate, at the forefront of the rights inquiry. Doing so would provide greater clarity as regards the scope of P1-3 and better align with the Court’s commitment to protecting and promoting democratic values.^93^ Democratic theorists have long opined that functioning and effective democracies require that the electorate has access to reliable and accurate information, both during and outside of elections.^94^ While P1-3 does not explicitly guarantee that voters must remain fully or accurately informed in pre-electoral periods, the repeated and systematic targeting of voters with false information arguably frustrates citizens’ meaningful and informed participation in electoral affairs.

## 3. *Recognising Proactive Positive Obligations*

The second limitation in the Court’s approach to P1-3, which can and should be rectified in *Bradshaw*, is the reliance on reactive positive obligations which come too late in the process to protect the right to free elections. The Court has recognised states’ positive obligation under P1-3 to effectively investigate election tampering. In *Bradshaw*, the applicants argue that this procedural obligation required the UK government to investigate hostile state interference in its domestic elections. Such a positive obligation (whether as existing or as the applicants have framed it) is inherently reactive. However, the applicants in *Bradshaw* have gone beyond this reactive requirement: they argue that P1-3 imposed an additional obligation on the UK government to implement an effective legal and institutional framework to secure its obligation to organise free elections under conditions which ensure the free expression of the opinion of the people. The recognition of such a proactive positive obligation would better protect the information environment during election periods and contribute towards a properly informed electorate.^95^ Such a proactive approach to states’ positive obligations would offer more robust protection for the rights of the electorate under P1-3 (discussed above) and better align with article 10 jurisprudence, including the Court’s articulation of states’ positive obligation to create a favourable environment for participation in public debate.^96^ We return to the relationship between P1-3 and article 10 in section 4.

This section canvasses the jurisprudence on states’ positive obligations under P1-3, before analysing its main drawback: such obligations have largely been framed reactively, around the duty to investigate voting irregularities or election tampering. We then illustrate the potential of *Bradshaw* to compel more robust—and proactive—state efforts in mitigating the spread of electoral disinformation and FIMI.

### A. Positive Obligations under P1-3

As noted above, the primary obligation flowing from P1-3 is a positive one: states must ‘undertake to hold free elections’ under specified criteria. The text makes express reference not only to how elections must occur at ‘reasonable intervals’ and through ‘secret ballot’, but also ‘under conditions which will ensure the free expression of the people in their choice of the legislature’.^97^ The ECtHR has held that this final clause requires that ‘elections cannot be conducted under any form of pressure in the choice of one or more candidates, and that in this choice the elector must not be unduly induced to vote for one party or another’.^98^ Owing to the broad language of P1-3, a wide range of violations have been found—including frequent modification of domestic electoral laws, non-provision of accessible voting procedures for applicants with physical disabilities and failure to ensure that the deregistration of electoral candidates was impartially adjudicated.^99^ As explained below, *Bradshaw* provides an opportune moment for the Court to recognise that a state’s failure to protect elections from disinformation and FIMI falls within the scope of P1-3 violations.

States’ positive obligations arise in both the *pre-election* period and the *post-election* period. In respect of the pre-election period, the Court affirmed in *Communist Party of Russia v Russia* that states have positive obligations under P1-3 in the context of media coverage of elections.^100^ Reiterating that there ‘can be no democracy without pluralism … which cannot be attained without the adoption of certain positive measures’, the Court held that the state had ‘an obligation to intervene in order to open up the media to different viewpoints’ in election periods.^101^ Stated differently, CoE states must ensure that coverage by state-controlled media is objective, balanced and ‘compatible with the spirit of “free elections”, even where no direct proof of deliberate manipulation was found’.^102^ In addition, the Court has held that where a system to eliminate or reduce inequality of political representation is effectuated, such measure ‘should contribute to the participation of national minorities on an equal footing with others in the choice of the legislature, rather than perpetuating the exclusion of minority representatives from political decision-making at a national level’.^103^

In respect of the post-election period, states’ obligations include: (i) ensuring careful regulation of the process in which voting results are ascertained, processed and recorded;^104^ (ii) establishing a system to ensure the effective examination of individual complaints and appeals in electoral matters;^105^ and (iii) investigating alleged electoral interference as part of their obligation to secure fair democratic elections.^106^ These post-election obligations are inherently reactive, comprising procedural and investigative obligations to determine what went wrong. By contrast, the pre-election obligations are more proactive and substantive. The requirement to ensure pluralistic debate is aimed at creating a favourable environment for public discourse during the crucial period where voters are informing themselves and preparing to cast their ballots. The requirement that legislation aimed at minority participation makes good on its objective is geared towards greater pluralism and diversity in political decision making. Critically, to the extent that voting irregularities and election tampering have been addressed, the Court has narrowed its scrutiny to states’ compliance with their *reactive, post-election* obligations while eschewing substantive inquiry into their *proactive* obligations to avoid elections becoming compromised in the first place.

### B. From Reactive Investigations to Proactive Obligations to Address FIMI

The positive obligations recognised under P1-3 do not go far enough in addressing the underlying conditions which are necessary to ensure the free expression of the opinion of the electorate. Instead, they are largely reactive: they arrive too late to meaningfully safeguard the quality, accuracy and reliability of information during election periods—all of which are of crucial importance for an informed electorate.^107^ While the obligation to ensure media pluralism in elections is more proactive—and more in line with creating the conditions necessary for free and informed elections—it remains unclear whether and to what extent such obligations compel states to take preventative action(s) to combat or mitigate the harms posed to public discourse by electoral disinformation and FIMI. This is both surprising and difficult to reconcile with the Court’s prior acknowledgement that states have a legitimate interest in ensuring that voters remain properly informed. The recognition and concrete development of pre-election obligations—specifically, upstream measures to ensure the quality and veracity of public discourse—and the recognition that post-election obligations extend to investigating credible claims of FIMI and disinformation are further areas where *Bradshaw* could make significant headway.

### C. *The Potential of* Bradshaw

The applicants in *Bradshaw* have alleged two violations of P1-3 by the UK government: first, the failure to launch an investigation into the credible claims of electoral disinformation and FIMI in previous elections; and second, the failure to implement an effective regulatory framework to secure free elections.

The first alleged failure invokes the states’ failure to comply with its (reactive) investigative obligations. Such obligations are well established in P1-3 jurisprudence, as discussed above. While the Court has not had occasion to consider whether retroactive procedural and investigative obligations apply to credible claims of disinformation and FIMI, such an extension of existing obligations would be consistent with its approach to vote tampering and electoral interference. As two domestic inquiries suggest credible evidence of Russian interference, the Court may accept that the UK had an obligation to investigate whether disinformation and FIMI skewed voter choice in the Scottish and EU referenda or the previous national election. Stated differently, P1-3 must surely require that, where credible reports reveal the weaponisation of disinformation and widespread FIMI in the pre-election period, the state must investigate the effects of the election interference where the ‘free choice’ of the electorate was potentially compromised. This would be a significant advancement in securing minimum standards for CoE states facing analogous electoral threats.

However, three practical hurdles would likely arise. First, such investigations would likely take considerable time to conclude, leaving voters and governments in a prolonged period of uncertainty (during which election results may be cast in doubt). Indeed, the lapse in time between the events in question in *Bradshaw* and the Court’s ultimate judgment may pose a significant challenge: Brexit has been negotiated and effected, and the national election result has been overtaken by a subsequent election in 2024. Second, establishing that disinformation and FIMI had a decisive impact on the election—whether on individual voter choice or on election outcomes—may prove an insurmountable hurdle. The impact of disinformation on election results is a topic of significant scholarly disagreement, and depends to a large extent on the standards of causation and proof that the Court will require.^108^ Third, even if an investigation concluded that disinformation and FIMI affected the election, it would (at best) be limited to offering lessons for future elections. While necessary, such an investigative obligation is not sufficient to ensure that the right to free elections is practical and effective, rather than theoretical and illusory.^109^ Upstream measures to safeguard political discourse and protect the exercise of voters’ free and informed choice are required.

The second alleged failure is thus where *Bradshaw* can most make its mark: the state’s obligation to implement an effective regulatory framework to secure free elections under conditions which ensure the free opinion of the people. This obligation is proactive, purposive and substantive. It is proactive because it requires the state to establish a framework to, *inter alia*, mitigate the harmful effects of electoral disinformation and FIMI on the underlying conditions necessary to ensure the free expression of the opinion of the people. It is purposive because it does not merely require *any* regulatory framework: it requires an *effective* regulatory framework—one that is both designed to safeguard the information environment in the run-up to elections and effectively achieves this aim in practice. It is substantive because it does not compel only compliance with procedural and formulaic requirements; to the contrary, such an obligation could (and should) illuminate what conditions are necessary to ensure the free opinion of the people, and what an ‘effective’ regulatory framework requires to ensure compatibility with P1-3.^110^ There are a range of measures which could be adopted to address the spread and harmful effects of electoral disinformation—from regulatory measures compelling the removal of disinformation online and sanctioning of individuals who disseminate it, to media and information literacy measures designed to empower critical engagement with information.^111^ States’ margin of appreciation is notably wide in respect of their obligations to hold free elections under P1-3, such that the Court may refrain from stipulating precise regulatory measures. And in practice, the nature and scale of the threat of FIMI may differ between CoE states, requiring some leeway in how they meet the challenge. However, guidance from the Court about minimum thresholds—or an acceptable range of options—that comply with states’ positive obligations under P1-3 would be a welcome advancement.

The shift towards proactive, or upstream, obligations under P1-3 would also be consistent with broader CoE initiatives to address disinformation and FIMI.^112^ For instance, in 2020, the Parliamentary Assembly of the Council of Europe (PACE) adopted Resolution 2326, entitled ‘Democracy hacked? How to respond?’.^113^ The resolution notes that in order to address disinformation challenges in the context of democratic elections, CoE states need to, *inter alia*, ‘enable voters to receive trustworthy information and become more informed and engaged, with a view to preserving the exercise of their right to truly free and fair elections’,^114^ and ‘consider updating national legislation in order to counter disinformation campaigns more effectively’.^115^ The PACE further calls on CoE states to implement strategies to ‘enable voters to evaluate critically electoral communication and increase society’s resilience to disinformation’.^116^ More recently, the Committee of Ministers of the CoE followed suit, adopting a recommendation on states’ positive obligations to address the threats posed by a polluted and toxic information environment. In a recommendation adopted in 2022, the Committee of Ministers adopted guidelines on promoting quality journalism in the digital age.^117^ The guidelines recognise that the spread of disinformation represents a growing threat to democracy, and that individuals’ trust in media as well as in politics, institutions and expertise has declined to ‘a worryingly low level’.^118^ The guidelines call for states’ full support of efforts to address disinformation and propaganda: most notably, they provide that, ‘[a] well-informed and media-literate society … is an essential part of the defence against information manipulation in democratic societies’.^119^

The PACE resolution and the Committee of Ministers recommendation recognise the critical importance of ‘fire-prevention’ initiatives alongside ‘fire-fighting’ efforts.^120^ The applicants in *Bradshaw* have asked the Court to follow suit, by recognising states’ proactive as well as reactive obligations in respect of electoral disinformation and FIMI. Of course, regulatory measures to address disinformation and FIMI may have corollary effects on the right to freedom of expression, recognised under article 10 ECHR. This leads us to the final opportunity provided by *Bradshaw*: clarifying the interplay between article 10 and P1-3 during elections.

## 4. *Reading Article 10 and P1-3 Harmoniously*

Contemporary debates surrounding disinformation and online communications reflect the tension between the need for an open and unencumbered information ecosystem, in which all information spreads freely, and the importance of a well-informed citizenry, equipped with accurate information, to enable democratic debate and decision making. The ECtHR has recognised analogous tensions in electoral settings: in previous cases, the ECtHR has indicated that P1-3 and article 10 are ‘inter-related and operate to reinforce each other’, including in respect of restrictions imposed during electoral campaigns.^121^ Although the applicants in *Bradshaw* have not raised an article 10 claim, the development of states’ positive obligations under P1-3 to address electoral disinformation and FIMI may have implications for expressive freedom. In particular, such measures may interfere with speakers’ rights to impart information and ideas in order to protect ‘the right of the public to be properly informed’.^122^ A regulatory framework which constrains public debate in order to combat electoral disinformation and FIMI may be consistent with states’ positive obligations under P1-3 and with states’ negative and positive obligations under article 10. However, the Court can—and should—recognise the potential impacts of such measures on article 10 rights and offer guidance about how P1-3 and article 10 can be interpreted harmoniously to ensure effective protections for speakers and audiences during election periods.

This section traces the Court’s commentary on the interplay between P1-3 and article 10 during elections, before examining the relevant jurisprudence on election falsehoods under article 10. We then distil the potential of *Bradshaw* to further elucidate the interplay of these rights in relation to electoral disinformation and, in so doing, ensure greater cohesion and clarity across the Convention framework.

### A. The Interplay of P1-3 and Article 10 in Pre-election Prohibitions on Expression

Freedom of expression protects speakers’ rights to impart information and ideas, as well as audiences’ rights to receive them. Both aspects of article 10 are crucial during elections; however, they may conflict where powerful speakers monopolise public discourse, where falsehoods proliferate or where foreign actors manipulate information in an effort to deceive the citizenry. The Court’s challenge, then, is determining where to draw the appropriate lines between speakers’ and audiences’ rights, between respecting individual rights and safeguarding the integrity of democratic processes. This tension is evident in the Court’s case law on statutory prohibitions on expression during pre-election periods.

The Court first recognised the symbiotic relationship between free elections and freedom of expression in *Bowman v United Kingdom*.^123^ The applicant distributed 25,000 anti-abortion leaflets prior to general elections, for which she was prosecuted for exceeding the statutory spending cap of five pounds to promote electoral candidates six weeks before an election.^124^ The Court reflected on the interplay between article 10 and P1-3 as follows:

Free elections and freedom of expression, particularly freedom of political debate, together form the bedrock of any democratic system. The two rights are inter-related and operate to reinforce each other: for example, as the Court has observed in the past, freedom of expression is one of the ‘conditions’ necessary to ‘ensure the free expression of the opinion of the people in the choice of the legislature’. For this reason, it is particularly important in the period preceding an election that opinions and information of all kinds are permitted to circulate freely.^125^

As this quotation makes clear, the rights to freedom of expression and to free elections may reinforce one another. However, the Court recognised that at times they conflict, such that it may be necessary in pre-election periods ‘to place certain restrictions, of a type which would not usually be acceptable, on freedom of expression, in order to secure the “free expression of the opinion of the people in the choice of the legislature”’.^126^ In *Bowman*, the applicant’s right to impart information conflicted with the need to protect free elections by ‘securing equality between candidates’.^127^ While the Court accepted that the latter was a legitimate aim, it reasoned that the five-pound spending cap constituted a ‘total barrier to Mrs Bowman’s publishing information with a view to influencing the voters … in favour of an anti-abortion candidate’.^128^ It thus violated article 10 ECHR.

The Court again alluded to the relationship between free elections and freedom of expression in *TV Vest AS & Rogaland Pensjonistparti v Norway*.^129^ The application challenged a statutory ban on televised political advertising; the first applicant, a television broadcaster, was fined for contravening the ban after it broadcast political advertisements for the second applicant, the Pensioners Party.^130^ Norway argued that the case was not about freedom of expression but ‘first and foremost the integrity of the democratic process and specifically the public’s—the voters’—right to fair democratic elections’.^131^ The government urged the Court to follow the ‘general reasoning’ in *Bowman* in relation to P1-3.^132^ The applicants, by contrast, argued that the ban disproportionately affected marginal political groups like the Pensioners Party, by effectively preventing them from communicating directly with the electorate on television.^133^ The Court recognised that the blanket ban sought to *inter alia* support the integrity of democratic processes.^134^ However, it held that the ban was disproportionate to the aim pursued, and an article 10 violation was found.^135^

In the more recent judgment of *Animal Defenders International v United Kingdom*, the Grand Chamber of the Court reached a different conclusion with respect to a statutory prohibition on paid political advertising on radio and television.^136^ The Grand Chamber did not expressly refer to P1-3, but attached considerable weight to the conclusions of UK parliamentary and judicial bodies that the ban ‘was necessary to prevent the distortion of crucial public interest debates and, thereby, the undermining of the democratic process’.^137^ Additionally, the Grand Chamber observed that the prohibition was ‘specifically circumscribed to address the precise risk of distortion the State sought to avoid with the minimum impairment of the right of expression’.^138^ Of critical importance in the Grand Chamber’s reasoning—and in several separate opinions—was the availability of alternative means for the applicant to communicate its views.^139^ This feature distinguished the case from *Bowman* and *TV Vest*. Accordingly, in *Animal Defenders*, the Grand Chamber held that the statutory prohibition was a proportionate interference with the applicant’s freedom of expression, as it safeguarded democratic debate from being distorted.^140^

The foregoing cases reveal that the Court has recognised the interplay between the rights to free elections and to freedom of expression and has sought to balance the protection of individual rights to receive and impart information under article 10 with the preservation of the integrity of democratic processes under P1-3. However, these judgments expose the difficulties inherent in undertaking such balancing—or in discerning from one case to the next how the balance between competing or conflicting rights will be struck.^141^ The Grand Chamber accepted that the political advertising ban in *Animal Defenders* was proportionate, though similar bans in *Bowman* and *TV Vest* were found to be disproportionate.^142^ While the Court is concerned with safeguarding the quality of pre-election discourse, it is unlikely to find proportionate interferences with article 10 that act as effective barriers to political expression.

While the above cases concern the need to protect public discourse *generally* during elections, similar tensions arise in cases of election *falsehoods* under article 10. Whereas the P1-3 jurisprudence on this topic arose in connection with electoral candidates’ inaccurate disclosures to electoral commissions, the article 10 case law concerns falsehoods by varying speakers—electoral candidates, political representatives, journalists and citizens—to the wider public. Here, too, the Court has construed the tension as a battle of individual rights, between the freedom of expression of the speaker and the right to reputation of the individual defamed. The public’s right to be properly informed—to receive truthful information during elections—has been overlooked.

### B. Article 10, False Electoral Information and the Public’s Right to Be Informed

The ECtHR has recognised the links between freedom of expression and democracy since its earliest article 10 judgments.^143^ In *Handyside v United Kingdom*, it held that ‘Freedom of expression constitutes one of the essential foundations’ of democratic society, as well as ‘one of the basic conditions for its progress’.^144^ Article 10 guarantees not only the freedom to express oneself, but also the freedom of the audience to receive such information—to be *informed*.^145^ The Court has, on occasion, recognised the importance of the public being provided accurate and truthful information, including during elections.^146^ For instance, in *Orlovskaya Irskra v Russia*, the applicant newspaper editors were prosecuted for publishing—during an election—articles accusing a governor of corruption.^147^ Referencing P1-3, the Court accepted that the relevant electoral law pursued the legitimate aim of ‘enforcing the voters’ right to impartial, truthful and balanced information via mass media outlets and the formation of their informed choices in an election’.^148^ However, in article 10 cases concerning electoral falsehoods, the Court’s focus shifts away from the macro (the electorate’s right to truthful information) and towards the micro (the rights to freedom of expression and to reputation of the individuals concerned). Far from balancing the rights to free elections and to freedom of expression, the contest is between the individual speaker’s right to spread falsehoods and the right of the individual targeted to his or her reputation. Indeed, the Court rarely refers to P1-3 in such cases (unless it is raised as a separate complaint by the applicant) despite its seeming relevance to the issues. Several cases are illustrative.

The first case of note is *Vitrenko and Others v Ukraine*, concerning alleged violations of article 10 and P1-3.^149^ The first applicant was the leader of the Progressive Socialist Party; the other applicants had been nominated to stand as candidates for her party in parliamentary elections. Ms Vitrenko was given an official warning by the Central Electoral Commission for false statements made during a live broadcast about her rival, contrary to electoral legislation.^150^ Her complaint to the Supreme Court about this warning was unsuccessful, as it found she had infringed electoral legislation by ‘publicly disseminating untrue statements’ about the leader of another political party.^151^ Over the same period, Ms Vitrenko’s registration as a candidate was cancelled for repeated violations of electoral legislation.^152^ Before the ECtHR, she challenged the restriction of her expression under article 10.^153^ The government did not contest the interference with Ms Vitrenko’s expressive freedom, but argued that it pursued, *inter alia*, ‘the public interest to ensure free and fair parliamentary elections’.^154^ Moreover, the government argued that the TV debate in question had been widely publicised and that ‘words used in an election campaign have particular weight and exert a great influence on the formation of public opinion’.^155^ The Court declared the application inadmissible. With respect to the article 10 claim, the Court held that Ms Vitrenko’s statements were not value judgments, but untrue statements of fact, communicated on a live broadcast in the absence of the person concerned.^156^ With respect to the remaining applicants’ P1-3 claim, the Court held that they had been ‘fully able to participate in the elections, having equal opportunities to other candidates’, and had ‘failed to show any appearance of a breach of their rights’ under P1-3.^157^ ‘The fact that they were unsuccessful,’ the Court explained, ‘does not amount to such a violation.’^158^

By contrast, in the more recent judgment in *Brzeziński v Poland*, the Court found an article 10 violation for sanctions imposed on the applicant, a municipal election candidate, for circulating campaign materials containing false information about his opponents.^159^ He had been prohibited from distributing the brochure, ordered to apologise and made to correct the inaccurate information in local daily newspapers.^160^ The ECtHR reiterated that article 10(2) leaves ‘little room for restrictions’ on political expression, and that the limits of permissible criticism are wider in respect of politicians than private individuals.^161^ Moreover, since the applicant was speaking as an opposition candidate, the state’s margin of appreciation was very limited.^162^ The Court accepted the necessity of ‘combat[ting] the dissemination of misleading information about election candidates in order to preserve the quality of public debate in the pre-election period’, but noted the importance during this period of allowing opinions and information of all kinds to circulate freely.^163^ Ultimately, the Court found that the domestic courts had failed to strike an appropriate balance between the applicant’s right to freedom of expression and the need to protect the rights and reputation of the complainants.^164^ The sanction imposed was likely to have an inhibiting effect on someone participating in political debate, and a violation of article 10 was therefore found.^165^

Finally, in *Staniszewski v Poland*, a journalist (and member of the municipality’s electoral commission) was sanctioned under the Election Code for publishing untrue statements about a candidate in local elections.^166^ The domestic courts held that the applicant’s newsletters contained demonstrably untrue statements about the incumbent mayor, designed to discourage the electorate from voting for him.^167^ Before the ECtHR, the applicant challenged the compatibility of the *Election Code* with article 10 ECHR.^168^ The government emphasised the particular context of safeguarding the integrity of electoral processes, and argued that the scheme was ‘necessary to protect a candidate during an election campaign from the dissemination of untrue information’.^169^ Though the Court did not explicitly invoke P1-3, it reiterated that free elections and freedom of expression are interrelated and stressed the importance of open political debate in the pre-election period.^170^ However, the Court proceeded to recognise ‘the importance of protecting the integrity of the electoral process from false information that affect[s] voting results, and the need to put in place the procedures to effectively protect the reputation of candidates’.^171^ Ultimately, the Court found no article 10 violation.^172^ The applicant had failed to either furnish evidence that his factual statements had been true or to challenge the domestic courts’ findings about their veracity. The domestic courts had appropriately balanced competing interests—the interest of participants in election campaigns to freely use all possible means to influence voters and the candidate’s right to be protected from untrue allegations.^173^

### C. From the Individual’s Rights to the Electorate’s Interests

Like the P1-3 jurisprudence canvassed above, the article 10 case law reveals the difficulties inherent in respecting speakers’ rights to freely impart information without restriction, on the one hand, and protecting the public’s right to be accurately informed. It also reflects the broader challenge of mediating individual rights while safeguarding democratic processes and the discursive practices which inform and sustain them.

While the ECtHR has reasoned that P1-3 and article 10 are interrelated but may conflict, it has not explained precisely *how* the two rights should be read together or how the operative distinctions in their text should inform such a reading. Three important distinctions between article 10 and P1-3 may bear on this analysis. First, like other substantive clauses in the Convention, article 10(1) provides that ‘Everyone has the right to freedom of expression’. By contrast, P1-3 stipulates that ‘The High Contracting Parties undertake …’.^174^ The Court has clarified that ‘the inter-State colouring of the wording of [P1-3] does not reflect any difference of substance from the other substantive clauses in the Convention and Protocols’.^175^ Instead, this framing was explained as follows:

The reason for it would seem to lie rather in the desire to give greater solemnity to the commitment undertaken and in the fact that the primary obligation in the field concerned is not one of abstention or non-interference, as with the majority of the civil and political rights, but one of adoption by the State of positive measures to ‘hold’ democratic elections.^176^

This relates to the second distinction: while article 10 entails negative and positive obligations, P1-3 is expressly about states’ positive obligations. The third and final distinction of relevance is the qualified nature of the rights. Article 10(1) is expressly qualified by article 10(2): restrictions are permissible where they satisfy the ‘triple test’ test of being prescribed by law, in pursuit of a legitimate aim and necessary in a democratic society.^177^ P1-3 contains no such express limitations; however, the Court has held that the rights in question ‘are not absolute’: ‘Since [P1-3] recognises them without setting them forth in express terms, let alone defining them, there is room for implied limitations.’^178^ The Court must, in each case, determine whether the requirements of P1-3 have been complied with, which requires satisfying itself that

the conditions do not curtail the rights in question to such an extent as to impair their very essence and deprive them of their effectiveness; that they are imposed in pursuit of a legitimate aim; and that the means employed are not disproportionate … In particular, such conditions must not thwart ‘the free expression of the opinion of the people in the choice of the legislature’.^179^

The ‘implied limitation’ concept means that the Court does not apply the traditional tests of ‘necessity’ or ‘pressing social need’, used in respect of article 10.^180^ Instead, it considers two criteria: first, whether there was arbitrariness or a lack of proportionality in the interference; and second, whether the restriction interfered with the free expression of the opinion of the people.^181^

Two issues relevant to the present inquiry thus arise from the jurisprudence. First, much of the article 10 jurisprudence (like the P1-3 jurisprudence) has developed primarily around individual *speakers’* rights; neither the audience’s right to be properly informed nor the integrity of the democratic process has been a key consideration or played a decisive role in the outcomes of such cases.^182^ There is a danger that in prioritising individual rights (to speak, to vote, to stand for election), the democratic rights of the populace are neglected in the jurisprudence. Such neglect may have serious consequences for the pillars supporting democracy—a properly informed electorate, an accurate and truthful exchange of information and ideas, and ultimately ‘the voting of wise decisions’.^183^ Second, while the Court has recognised the interplay between article 10 and P1-3, this recognition has been more rhetorical than substantive. Stated differently, it remains unclear how states may fulfil P1-3 positive obligations to ensure the ‘conditions necessary for the free expression of the opinion of the people’ without undermining their negative obligations (of restraint) under article 10. The Court has provided little analytical guidance on how these rights can be read harmoniously, particularly where they conflict.

### D. *The Potential of* Bradshaw

It is in respect of these two issues that *Bradshaw* could have a meaningful impact: first, in restoring the balance between individual rights and collective rights; and second, in providing guidance on how the rights to freedom of expression and free elections should be read together where they conflict.

On the first point: article 10 protects not only speakers’ rights to impart information and ideas, but also the public’s right to receive them, to be informed. However, the jurisprudence has largely developed around *speakers’* rights; the audience’s right to be properly informed has been recognised only obliquely, where the press’s right to impart information has been restricted.^184^ The audience’s right is rarely foregrounded in such cases, meaning that the scope and limits of this branch of article 10 have not developed to the same extent as speakers’ rights to impart information and ideas.^185^ This differentiation may be consequential in the context of electoral disinformation and FIMI. The Court has repeatedly stressed that political expression must be afforded a heightened level of protection under article 10—thus, measures taken by states to impede speakers’ dissemination of information and ideas will be subjected to significant scrutiny.^186^ Less clear, however, is how the right of the public to receive political expression is to be weighed in the balance—for instance, whether the electorate’s right to receive accurate and reliable information (or to be protected against inaccurate information) could ever take precedence in such a contest.^187^

*Bradshaw* is distinguishable from the existing case law in an important respect: the applicants’ individual rights (to speak, to vote, to stand for election, or even to their reputations) are not at stake. Instead, the applicants argue that the state has forsaken its obligations to the broader public to foster the conditions necessary for free elections, which include regulating electoral disinformation and FIMI. This puts the less-developed aspect of freedom of expression—the electorate’s right to receive accurate information, to be properly informed—at the forefront of the rights inquiry. Of course, the application concerns the conditions necessary for free expression *in elections*; it is only a P1-3 claim, rather than an article 10 claim, that has been advanced. However, Eric Heinze has argued that voting is nothing more than ‘a formalized procedure for speaking’; vote and voice should be conceived as ‘two manifestations of the same legitimating expressive condition’ of democracy.^188^  *Bradshaw* can expand the lens beyond individual speakers or individual voters, to encompass the collective democratic rights of the electorate. Doing so would better safeguard the broader purposes underpinning the rights to free elections and freedom of expression: a vigorous but informed democratic debate which enables and sustains self-government.^189^

On the second point, *Bradshaw* affords the Court the opportunity to explain how P1-3 and article 10 are to be read harmoniously, despite their aforementioned textual and interpretive differences. The applicants in *Bradshaw* have argued that the UK failed in its positive obligation to implement an effective regulatory framework to secure its obligations to hold free elections. Should the Court agree, an ‘effective’ framework may well require certain interventions into the ‘marketplace of ideas’^190^ to mitigate FIMI, including electoral disinformation. This may place the rights protected by P1-3 and article 10 on a collision course, particularly if the Court adopts a narrow interpretation of *speakers’—*as opposed to *audiences’—*expressive freedoms.

The Court can, and should, recognise the dangers to expressive freedom that such measures may pose. In addition, the Court can, and should, provide guidance on how states can fulfil their positive obligations under P1-3 to ensure the ‘conditions necessary for the free expression of the opinion of the people’ without contravening their article 10 (negative or positive) obligations. To this end, the Court has identified several positive obligations under article 10 which concern the trustworthiness of information conveyed to the public by the media and public authorities, and the importance of safeguarding the information environment for democratic participants. For instance, in *NIT SRL v Moldova*, the Court accepted that ‘the need to preserve the public’s access to impartial, trustworthy and diverse political speech through television news programmes was at the heart of the national authorities’ decision to uphold the sanction imposed’.^191^ In *Association Burestop 55 and Others v France*, the Court held that ‘respect for the right of access to information necessarily implies that the information provided [by the public authority] must be reliable, in particular where this right results from a legal obligation imposed on the State’.^192^ Thus, ‘the provision of insincere, inaccurate or inadequate information in such circumstances is tantamount to a refusal to inform’.^193^ Moreover, the Court has occasionally reaffirmed that states are under a positive obligation to create a favourable environment for participation in public debate without fear by all persons concerned.^194^ If extended and refined, these positive obligations could have relevance for electoral disinformation and FIMI. As Andrew Kenyon posits:

Democracy entails positive freedom of communication, not only a negative freedom or liberty of speech. This means the state has obligations to act in support of communicative freedom as well as there being limits on state restriction of speech; both positive and negative dimensions of free speech need protection for democracy to have substantial communicative legitimacy.^195^

The same could be said about free elections, as the applicants in *Bradshaw* have argued (and as the Romanian Constitutional Court recently held). The Court should take this opportunity to recognise states’ positive obligations to act in support of informed electoral debate, to safeguard the ‘voting of wise decisions’ and to resist the effects of disinformation and FIMI on the free expression of the people’s opinion. In doing so, the Court can illuminate the interrelationship between P1-3 and article 10, and provide guidance on how states can properly mediate democratic and expressive rights, particularly where they conflict.

## 5. *Conclusion*

With *Bradshaw*, the ECtHR may, for the first time, recognise that election disinformation and FIMI interfere with the right to free elections and elucidate states’ obligations to mitigate their harmful effects. The case could thus be a seminal one, with repercussions throughout CoE states. The nature of the application, in particular its focus on the public’s democratic rights, presents a unique opportunity for the Court to refine and reformulate its approach to free elections under P1-3 of the Convention. Doing so has never been more imperative, in light of conflicting domestic judgments about whether and to what extent states must act in the face of credible threats of FIMI and electoral disinformation. This article has contended that the Court is now at a crossroads: with *Bradshaw*, it can address existing shortcomings and recurring ambiguities that have taken hold in the Court’s interpretation of the right to free elections under the Convention or it can further entrench these doctrinal limitations. While there will undoubtedly be much scholarly critique following the release of *Bradshaw* in the coming years, such perennial shortcomings warrant proactive scholarly debate and consideration to inform the Court’s reasoning before it is too late.

At this critical juncture, this article has identified and critiqued three limitations in the Court’s free elections case law. It has offered analytical pathways to address each in turn to meaningfully protect democratic elections and democratic rights. First, the Court should shift away from the individualised approach to P1-3 which has defined its free elections jurisprudence. While the recognition of individual rights to vote and to stand for election is necessary, it is not sufficient to ensure the right to free elections. Something has been lost in translating the positive rights and public purposes of P1-3—for the benefit of the electorate—into individual candidates’ claims before Strasbourg. The Court has the opportunity to foreground the rights of voters as democratic participants in *Bradshaw* (what we have termed the ‘public aspect’ of P1-3), which would offer stronger and more comprehensive protection for the citizenry’s democratic rights. Second, the Court should recognise and develop states’ proactive positive obligations to combat electoral disinformation and FIMI, rather than relying on reactive measures after the right to free elections has been undermined. This would not only ensure meaningful upstream protection for electoral processes, but provide guidance to CoE states and regional organisations about the measures they must implement to ensure the free expression of voters’ choices in democratic processes. Finally, the Court should clarify the relationship between the rights to free elections under P1-3 and to freedom of expression under article 10, particularly where they come into conflict. Doing so would provide greater coherence within and across the Convention system and would offer instructive guidance to states about how they can fulfil their institutional (positive) obligations under P1-3 while complying with their obligations in respect of article 10.

Election disinformation and FIMI remain significant concerns for states as well as regional organisations such as the European Union and the CoE. The spread of false information has been identified as a foremost risk for the global community and a constituent part of the current ‘information crisis’.^196^ As states and regional actors intensify efforts to combat election disinformation and FIMI, it is imperative that they do so in full compliance with their human rights obligations and in a manner which safeguards democratic values. With *Bradshaw*, the Court has an opportunity to shed light on what CoE states can (and must) do to combat election disinformation and FIMI in accordance with their obligations to hold free and fair elections, while complying with their obligations to protect expressive freedoms. This article has outlined the pitfalls to be avoided in doing so and provided concrete and principled suggestions for the path forward.

## Footnotes

1 Disinformation refers to ‘information that is false and deliberately created to harm a person, social group, organization or country’: see Claire Wardle and Hossein Derakhshan, ‘Information Disorder: Toward an Interdisciplinary Framework for Research and Policy Making’ (Council of Europe report DGO(2017)09 2017) <https://edoc.coe.int/en/media/7495-information-disorder-toward-an-interdisciplinary-framework-for-research-and-policy-making.html> accessed 29 November 2023.

2 Marta Pérez-Escolar, Darren Lilleker and Alejandro Tapia-Frade, ‘A Systematic Literature Review of the Phenomenon of Disinformation and Misinformation’ (2023) 11 Media and Communication 76; Rachel Kuo and Alice Marwick, ‘Critical Disinformation Studies: History, Power, and Politics’ [2021] Harvard Kennedy School Misinformation Review <misinforeview.hks.harvard.edu/article/critical-disinformation-studies-history-power-and-politics/> accessed 12 October 2024; Deen Freelon and Chris Wells, ‘Disinformation as Political Communication’ (2020) 37 Political Communication 145.

3 Tatiana Dourado, ‘Who Posts Fake News? Authentic and Inauthentic Spreaders of Fabricated News on Facebook and Twitter’ (2023) 17 Journalism Practice 2103; Soroush Vosoughi, Deb Roy and Sinan Aral, ‘The Spread of True and False News Online’ (2018) 359 Science 1146.

4 See eg Regulation (EU) 2022/2065 of the European Parliament and of the Council of 19 October 2022 on a Single Market For Digital Services and amending Directive 2000/31/EC (Digital Services Act) (Text with EEA relevance) [2022] OJ L277/1.

5 Rebecca K Helm and Hitoshi Nasu, ‘Regulatory Responses to “Fake News” and Freedom of Expression: Normative and Empirical Evaluation’ (2021) 21 Human Rights Law Review 302; Rachael Craufurd Smith, ‘Fake News, French Law and Democratic Legitimacy: Lessons for the United Kingdom?’ (2019) 11 JML 52. See also Elena Abrusci, ‘The UK Online Safety Act, the EU Digital Services Act and Online Disinformation: Is the Right to Political Participation Adequately Protected?’ (2024) 16 JML 440.

6 This article contributes to this expanding scholarship. See also Katie Pentney, ‘States’ Positive Obligation to Create a Favourable Environment for Participation in Public Debate: A Principle in Search of a Practical Effect?’ (2024) 16 JML 146; Ethan Shattock, ‘Free and Informed Elections? Disinformation and Democratic Elections Under Article 3 of Protocol 1 of the ECHR’ (2022) 22 Human Rights Law Review 1.

7 Convention for the Protection of Human Rights and Fundamental Freedoms, as amended, Council of Europe Treaty Series No 5, art 3 of Protocol 1 (ECHR).

8 Richard Nash, ‘The Romanian 2024 Election Annulment: Addressing Emerging Threats to Electoral Integrity’ (International Foundation for Electoral Systems, 20 December 2004) <ifes.org/publications/romanian-2024-election-annulment-addressing-emerging-threats-electoral-integrity> accessed 2 February 2025.

9 Romanian Constitutional Court Decision No 32 (6 December 2024) 10.

10 ibid.

11
*Bradshaw and Others v United Kingdom (communicated)* [2022] ECtHR 15653/22. The applicants are MPs from the Labour, Green and Scottish National parties.

12 ibid 11–14.

13
*Bradshaw* (n 11). The far-right presidential candidate in the Romanian elections also brought an application before the ECtHR, which was declared inadmissible: *Călin Georgescu v Romania (dec)* [2025] ECtHR 37327/24.

14 Although the Court has commented on election interference issues without expressly using the term ‘disinformation’ in its jurisprudence, as explained below.

15 Digital, Culture, Media and Sport Committee, *Disinformation and ‘Fake News’: Final Report* (2019, HC 1791) para 273. The Committee defines disinformation as ‘the deliberate creation and sharing of false and/or manipulated information that is intended to deceive and mislead audiences, either for the purposes of causing harm, or for political, personal or financial gain’: ibid para 3.

16 The final published report is available at <hansard.parliament.uk/Commons/2020-07-22/debates/8FB95D9A-A489-4F3C-8840-017A7A00949A/IntelligenceAndSecurityCommitteeRussiaReport>.

17 Intelligence and Security Committee of Parliament, *Russia* (2020, HC 632) para 12 <isc.independent.gov.uk/wp-content/uploads/2021/01/20200721_HC632_CCS001_CCS1019402408-001_ISC_Russia_Report_Web_Accessible.pdf>.

18 ibid 33.

19
*Bradshaw* (n 11) para 5. An additional ground for judicial review related to the Prime Minister’s public law duties, which was not challenged before the ECtHR and is not addressed further here.

20 ibid 7–9.

21 ibid 11–14.

22 ibid 15.

23 ‘MPs’ Landmark Legal Action against UK Government over Russian Interference Proceeds in European Court of Human Rights’ (Leigh Day, 19 January 2023) <leighday.co.uk/news/news/2023-news/mps-landmark-legal-action-against-uk-government-over-russian-interference-proceeds-in-european-court-of-human-rights/>.

24 Lengthy delays have been a point of scholarly critique: see eg Lewis Graham, ‘Boldness, Caution, Avoidance: Recent Cases Against the UK Before the European Court of Human Rights’ (2024) 5 European Convention on Human Rights Law Review 65, 79–80 (noting the ‘generally slow processing of applications’ in respect of the UK, with some taking nine years to resolve).

25
*Bowman v United Kingdom* [1998] ECtHR [GC] 24839/94. See Rubén Marciel, ‘On Citizens’ Right to Information: Justification and Analysis of the Democratic Right to Be Well Informed’ (2023) 31 Journal of Political Philosophy 358.

26 Paula Erizanu, ‘Romania’s “Rigged” Election Shows Europe the Dangers of Russian Disinformation’ *The Guardian* (11 December 2024) <theguardian.com/commentisfree/2024/dec/11/romania-presidential-election-russia-disinformation-europe> accessed 12 December 2024.

27 Rory O’Connell, ‘Realising Political Equality: The European Court of Human Rights and Positive Obligations in a Democracy Special Issue: Positive Obligations and the European Court of Human Rights’ (2010) 61 NILQ 263. Spurring this development was *Mathieu-Mohin and Clerfayt v Belgium* [1987] ECtHR 9267/81.

28 See eg *Mathieu-Mohin and Clerfayt* (n 27) paras 48–51; *Zdanoka v Latvia* [2006] ECtHR [GC] 72794/01 [102]; *X v Federal Republic of Germany (dec)* [1960] European Commission 530/59; *X and Others v Belgium (dec)* [1961] European Commission 1065/61; *X v Belgium* [1961] European Commission 1028/61. See also Javier García Roca, ‘From States’ International Commitment to Organise Free Elections to the Citizens’ Right to Vote and Stand for Election (Art 3 P1 ECHR)’ in Javier García Roca and Pablo Santolaya (eds), *Europe of Rights: A Compendium on the European Convention of Human Rights* (Brill 2012).

29 Agustin Ruiz Robledo, ‘The Construction of the Right to Free Elections by the European Court of Human Rights’ (2018) 7 Cambridge International Law Journal 225.

30
*Sarukhanyan v Armenia* [2008] ECtHR 38978/03.

31 ibid.

32 ibid 42.

33 ibid 42, 49.

34 ibid 49.

35 See also *Russian Conservative Party of Entrepreneurs and Others v Russia* [2007] ECtHR 55066/00, 55638/00.

36
*Sarukhanyan* (n 30) para 94.

37 ibid.

38
*Krasnov and Skuratov v Russia* [2007] ECtHR 17864/04, 21396/04.

39 ibid 44.

40 ibid.

41 ibid 48.

42 ibid.

43 ibid 49–51.

44 ibid 50.

45 ibid 60.

46 ibid 61.

47 ibid 63.

48 ibid 51, 67.

49 Paolo Cavaliere, ‘The Truth in Fake News: How Disinformation Laws Are Reframing the Concepts of Truth and Accuracy on Digital Platforms’ (2022) 3 European Convention on Human Rights Law Review 481.

50
*Melnychenko v Ukraine* [2004] ECtHR 67100/01, 69324/01, 73643/01.

51 ibid 20.

52 ibid 46, 65.

53 See generally Jacob Rowbottom, ‘Lies, Manipulation and Elections—Controlling False Campaign Statements’ (2012) 32 OJLS 507.

54 This distinction was drawn in the recent request for interim measures in *Georgescu* (n 13). The far-right candidate brought an application before the ECtHR alleging, *inter alia*, a violation of P1-3, and requested interim measures ‘in order to prevent irreparable harm to the democratic rights of the applicant *and Romanian citizens*’ (emphasis added): see ECtHR Press Release, ‘No Interim Measures Issued against Annulment of Romanian Presidential Election’ (21 January 2025) <hudoc.echr.coe.int/eng-press?i=003-8138373-11400351> accessed 2 February 2025.

55
*Babenko v Ukraine (dec)* [1999] ECtHR 30256/15 and others.

56 ibid.

57 ibid.

58
*Kerimova v Azerbaijan* [2010] ECtHR 20799/06 [11]–[12], [48].

59 ibid 51.

60 ibid 41.

61
*Davydov and Others v Russia* [2017] ECtHR 75947/11 [8].

62 ibid 20, 45, 247.

63 ibid 333.

64
*Mugemangango v Belgium* [2020] ECtHR [GC] 310/15.

65 ibid 123.

66 ibid 79, 83.

67 Vladislava Stoyanova, *Positive Obligations under the European Convention on Human Rights: Within and Beyond Boundaries* (OUP 2023) 116–17.

68
*Davydov* (n 61) para 285.

69
*Zdanoka* (n 28) para 103.

70 ibid 100.

71
*The Sunday Times v United Kingdom (no 1)* [1979] ECtHR 6538/74 [65]–[66].

72
*Staniszewski v Poland* [2021] ECtHR 20422/15 [47], citing *Mathieu-Mohin and Clerfayt* (n 27) para 47.

73 Richard Moon, *The Constitutional Protection of Freedom of Expression* (University of Toronto Press 2000) 41; Andrew T Kenyon, *Democracy of Expression: Positive Free Speech and Law* (1st edn, CUP 2021) 4.

74 Marciel (n 25) 1.

75 ibid.

76
*Zdanoka* (n 28) para 104, citing *Hirst v United Kingdom (no 2)* [2005] ECtHR [GC] 3282/12 and others [62].

77 See also O’Connell (n 27).

78 The right is framed positively and stands apart from other provisions in the Convention for this reason, as explained in more detail below.

79 United Nations, ‘Universal Declaration of Human Rights’ <www.un.org/en/about-us/universal-declaration-of-human-rights> accessed 3 February 2025.

80 ECtHR, Preparatory work on Article 3 of Protocol No 1 to the European Convention on Human Rights, Strasbourg, 5 September 1986 <echr.coe.int/documents/d/echr/echrtravaux-p1-3-cour-86-36-bil1221606> accessed 2 February 2025. Various amendments were put forward to remove this clause, and/or to replace it with a reference to a government which ‘represent[s] the people’ (the UK delegation argued for its deletion on the basis the phrase ‘is a rather misleading over-simplification’ and ultimately vetoed its inclusion in the original Convention within the Committee of Ministers: ibid 5, 8–9, 45–7, 52–3). See also Robledo (n 29).

81 This modified formulation reflected the concern that the original text ‘might be interpreted to require the institution of some form of proportional representation’: ECtHR Preparatory work (n 86) 74.

82 ECHR, arts 34 and 35(3)(b). The latter provides the following exception: ‘unless respect for human rights as defined in the Convention and the Protocols thereto requires an examination of the application on the merits’.

83 See eg *Le Mailloux v France (dec)* [2020] ECtHR 18108/20 [11].

84
*Bradshaw* (n 11) Questions to the Parties, Question 4.

85
*Verein Klimaseniorinnen Schweiz and Others v Switzerland* [2024] ECtHR [GC] 53600/20.

86 ibid 179–99.

87 More recently, the Court has ‘made clear that this recognition of standing of associations was justified by “specific considerations relating to climate change” … and limited to “this specific context”’: *Cannavacciuolo and Others v Italy* [2025] ECtHR 51567/14, 39742/14, 74208/14, 21215/15 [220]–[222], citing *Verein Klimaseniorinnen Schweiz and Others v Switzerland* (n 85) paras 498–9.

88
*Mugemangango* (n 64), citing *Hirst (no 2)* (n 76) para 61; *Zdanoka* (n 28) para 103.

89 Alain Zysset, ‘Calibrating the Response to Populism at the European Court of Human Rights’ (2022) 20 ICON 976, 1000.

90 For instance, the ‘living instrument’ doctrine and construction of rights that are not ‘theoretical or illusory but rights that are practical and effective’: see *Tyrer v United Kingdom* [1978] ECtHR 5856/72 [31]; *Airey v Ireland* [1979] ECtHR 78103/14 [24]. See also George Letsas, ‘The ECHR as a Living Instrument: Its Meaning and Legitimacy’ in Andreas Føllesdal, Birgit Peters and Geir Ulfstein (eds), *Constituting Europe* (1st edn, CUP 2013).

91
*Mugemangango* (n 64) para 73 (and citations therein).

92 See Request for interim measures refused concerning Romania 21 January 2025 <echr.coe.int/w/request-for-interim-measures-refused-concerning-romania>. See also Venice Commission, Opinion No 1218/2024, 27 January 2025 <www.venice.coe.int/webforms/documents/default.aspx?pdffile=CDL-PI(2025)001-e>.

93 See eg the Convention preamble.

94 See eg Marciel (n 25). See also Robert Dahl, ‘A Democratic Dilemma: System Effectiveness versus Citizen Participation’ (1994) 109 Political Science Quarterly 23; Douglas Kellner, ‘Habermas, the Public Sphere, and Democracy’ in Diana Boros and James Glass (eds), *Reimagining Public Space* (Palgrave Macmillan 2014) 19–43; Toril Aalberg and James Curran (eds), *How Media Inform Democracy: A Comparative Approach* (Routledge 2012).

95 Analogous points have been raised by third party interventions in *Bradshaw*: see eg ‘LSE Law Students Intervene on Social Media and Elections ECHR Case’ (London School of Economics, 24 March 2023) <lse.ac.uk/law/news/2023/echr>.

96
*Dink v Turkey* [2010] ECtHR 2668/07; see also Pentney (n 6).

97 ECHR P1-3.

98
*Bakirdzi and E.c v Hungary* [2022] ECtHR 49636/14, 65678/14 [43].

99 See *Petkov and Others v Bulgaria* [2009] ECtHR 77568/01, 178/02, 505/02; *Tănase and Chirtoacă v Moldova* [2008] ECtHR 7/08; *Toplak and Mrak v Slovenia* [2021] ECtHR 34591/19, 42545/19.

100
*Communist Party of Russia and Others v Russia* [2012] ECtHR 29400/05 [124]–[126].

101 ibid 126.

102 ibid 123.

103
*Bakirdzi* (n 98) para 73.

104
*Davydov* (n 61) paras 284–5.

105 See eg *Namat Aliyev v Azerbaijan* [2010] ECtHR 18705/06 [81]; *Uspaskich v Lithuania* [2016] ECtHR 14737/08 [93].

106 See eg *Babenko* (n 55).

107 Katie Pentney and Tarlach McGonagle, ‘From Risk to Reward? The DSA’s Risk-Based Approach to Disinformation’ in Maja Cappello (ed), *Unravelling the Digital Services Act package* (European Audiovisual Observatory (Council of Europe) 2021) 48.

108 See eg Dave Karpf, ‘How Digital Disinformation Turned Dangerous’ in Steven Livingston and W Lance Bennett (eds), *The Disinformation Age* (CUP 2020) 161–2; Hunt Allcott and Matthew Gentzkow, ‘Social Media and Fake News in the 2016 Election’ (2017) 32 Journal of Economic Perspectives 211. See also Sacha Altay and Alberto Acerbi, ‘People Believe Misinformation Is a Threat Because They Assume Others Are Gullible’ (2023) 26 New Media & Society 6440, 2.

109
*Airey* (n 90) para 24.

110
*Bakirdzi* (n 98) Joint Concurring Opinion of Judges Bošnjak and Derenčinović [3] (‘a violation of the Protocol will be found only in cases where the freedom of choice of the voters or the secrecy of the ballot were at stake’).

111 Pentney (n 6) 173–5.

112 It would also be consistent with the development of analogous positive obligations under art 10, including states’ obligation to create a favourable environment for participation in public debate by all persons without fear: *Dink* (n 96); Pentney (n 6).

113 ‘Democracy Hacked? How to Respond?’ (Parliamentary Assembly of the Council of Europe 2020) Resolution 2326. Notably, the Court referred to the PACE resolution in communicating *Bradshaw* to the UK: see *Bradshaw* (n 11) paras 41–2.

114 ibid 5.2.

115 ‘Democracy Hacked? How to Respond?’ (n 113) para 5.4.

116 ibid 6.1.

117 Recommendation CM/Rec(2022)4 of the Committee of Ministers to member States on promoting a favourable environment for quality journalism in the digital age (adopted by the Committee of Ministers on 17 March 2022 at the 1429th meeting of the Ministers’ Deputies).

118 ibid Guidelines [6]–[7].

119 ibid [2.5.4].

120 Pentney (n 6) 171–2.

121
*Bowman* (n 25). See also *Selahatt**i̇**n Dem**i**˙rta**ş*  *v Turkey (no 2)* [2020] ECtHR [GC] 14305/17 [392].

122
*Sunday Times (no 1)* (n 71) paras 65–6.

123
*Bowman* (n 25).

124 ibid 13.

125 ibid 42, citing *Mathieu-Mohin and Clerfayt* (n 27) paras 47, 54; see also *Lingens v Austria* [1986] ECtHR 9815/82 [41]–[42].

126
*Bowman* (n 25) para 43.

127 ibid 47.

128 ibid.

129
*Tv Vest as and Rogaland Pensjonistparti v Norway* [2008] ECtHR 21132/05 [65].

130 ibid 63.

131 ibid 41.

132 ibid 50.

133 ibid 30.

134 ibid 70.

135 ibid 71–8.

136
*Animal Defenders International v United Kingdom* [2013] ECtHR [GC] 48876/08 [76]. cf *Verein Gegen Tierfabriken Schweiz (vgt) v Switzerland (no 2)* [2009] ECtHR [GC] 32772/02.

137
*Animal Defenders* (n 136) para 116. The Grand Chamber acknowledged that the risk to pluralist public debates and free elections is not confined to electoral periods, ‘since the democratic process is a continuing one to be nurtured at all times by a free and pluralist public debate’: ibid 111.

138 ibid 117.

139 ibid 117, 124, Concurring Opinion of Judge Bratza [5]; Joint Dissenting Opinion of Judges Ziemele, Sajó, Kalaydjieva, Vučinić and De Gaetano [13].

140 ibid 125.

141 On the tensions between individual freedoms reflected in arts 8–11 ECHR and states’ positive electoral duties under P1-3, see Bosko Tripkovic and Alain Zysset, ‘Uncovering the Nature of ECHR Rights: An Analytical and Methodological Framework’ (2024) 24 Human Rights Law Review 1.

142 See also *VGT* (n 136).

143
*Handyside v United Kingdom* [1976] ECtHR 5493/72; *Sunday Times (no 1)* (n 71).

144
*Handyside* (n 143) para 49.

145 In *Sunday Times (no 1)*, the Court recognised that art 10 protects the public’s right to be *properly* informed—a qualifier it has dropped in subsequent cases: see *Sunday Times (no 1)* (n 71) para 66; but see the Dissenting Opinion of Judge Herndl in *Verlagsgruppe News Gmbh v Austria (no 2)* [2006] ECtHR 10520/02 (‘the Court has forcefully endorsed, in numerous judgments, the idea that the general public has a right to be properly informed—a right which is indeed essential in a democratic society’). See also Marciel (n 25).

146 For instance, the Court recently held that ‘the right of access to information would be deprived of its substance if the information provided by the competent authorities was insincere, inaccurate or even insufficient’: see *Association Burestop 55 and Others v France* [2021] ECtHR 56176/18 and others [108] (author’s translation); see also Katie Pentney, ‘The Right of Access to “Reliable” Information Under Article 10 ECHR: From Meagre Beginnings to New Frontiers’ (2024) 5 European Convention on Human Rights Law Review 230.

147
*Orlovskaya Iskra v Russia* [2017] ECtHR 42911/08.

148 ibid 104. On the facts, however, the Court found an art 10 violation because of overbreadth. See also *Ooo Informatsionnoye Agentstvo Tambov-Inform v Russia* [2021] ECtHR 43351/12 [81]–[92].

149
*Vitrenko and Others v Ukraine (dec)* [2008] ECtHR 4035/08.

150 ibid 3–4.

151 ibid 2–4. She referred to her rival as a ‘thief’ who had enabled the former Prime Minister to ‘rob’ Ukraine and stated that her rival would ‘never wash out her guilt’: ibid 2.

152 ibid 6.

153 ibid 9.

154 ibid 10.

155 ibid.

156 ibid 12, citing *Fuentes Bobo v Spain* [2000] ECtHR 63608/00 [46].

157
*Vitrenko* (n 149) 14.

158 ibid, citing *Babenko* (n 55).

159
*Brzezi* *ń* *ski v Poland* [2019] ECtHR 47542/07.

160 ibid 16.

161 ibid 53.

162 ibid 54, citing *Jean-Jacques Morel v France* [2013] ECtHR 25689/10 [39].

163 ibid 55 (authors’ translation), citing *Kwiecie**ń*  *v Poland* [2007] ECtHR 51744/99.

164
*Brzezi* *ń* *ski* (n 159) paras 58–60.

165 ibid 60–3; a similar result was reached in *Kość v Poland* [2017] ECtHR 34598/12.

166
*Staniszewski* (n 72).

167 ibid 20–1, 26.

168 ibid 38.

169 ibid 41. The Court accepted that the interference had pursued the legitimate aim of protecting election integrity and ‘the rights of the voters’: ibid 44.

170 ibid 47, citing *Mathieu-Mohin and Clerfayt* (n 27) para 47; *Bowman* (n 25) para 42.

171
*Staniszewski* (n 72) para 47, citing *Brzezi**ń**ski* (n 159) para 35.

172
*Staniszewski* (n 72) para 58.

173 ibid 52–3.

174
*Mathieu-Mohin and Clerfayt* (n 27) para 48.

175 ibid 50.

176 ibid. The *travaux préparatoires* refer throughout to P1-3 under the heading of ‘Political Rights’; however, late in the drafting process, the heading changed to ‘Free Elections’: see ECtHR Preparatory work (n 86).

177 J Gerards, ‘How to Improve the Necessity Test of the European Court of Human Rights’ (2013) 11 ICON 466; Tripkovic and Zysset (n 141) (making a similar observation in respect of art 9 and P1-3).

178
*Mathieu-Mohin and Clerfayt* (n 27) para 52.

179 ibid.

180
*Etxeberria Barrena Arza Nafarroako Autodeterminazio Bilgunea and Aiarako and others v Spain* [2009] ECtHR 35579/03, 35613/03, 35626/03, 35634/03 [47]–[50], cited with approval in *Eusko Abertzale Ekintza—Accion Nationalista Vasca (eae-Anv) c Spain* [2010] ECtHR 51762/07, 51882/07 [51].

181
*Etxeberria* (n 180) paras 47–50; *Eusko Abertzale Ekintza* (n 180) para 51.

182
*Zdanoka* (n 28) para 104, citing *Hirst (no 2)* (n 76) para 62. The ECHR’s strict standing requirements have likely played a role here too, as in the P1-3 jurisprudence canvassed above.

183 Alexander Meiklejohn, *Political Freedom* (OUP 1965) 27, 75. Galston puts the matter differently: ‘Democratic government, we have seen, needs truth to do its business well. It also needs truth to justify the power it wields. The alternatives to truth as the basis of democratic legitimacy are unattractive’: William A Galston, ‘Truth and Democracy: Theme and Variations’ in Jeremy Elkins and Andrew Norris (eds), *Truth and Democracy* (University of Pennsylvania Press 2012) 144. See also Sophia Rosenfeld, *Democracy and Truth: A Short History* (University of Pennsylvania Press 2019) 1–3.

184 See eg *Sunday Times (no 1)* (n 71).

185 This is perhaps most evident in access to information cases: the art 10 jurisprudence has developed around the right of intermediary ‘watchdogs’, such as the press and civil society organisations, to impart information and ideas, which requires access to information. The right of the public to receive it is alluded to as a secondary point, rather than the primary purpose of art 10. See eg *Magyar Helsinki Bizottság v Hungary* [2016] ECtHR [GC] 18030/11.

186
*Teslenko and Others v Russia* [2022] ECtHR 49588/12 and others [133] (‘The Court considers that calls to abstain from voting in an election are an instance of political expression … and thus, in principle, fall within the scope of expression that should be afforded the heightened level of protection under Article 10’: ibid).

187 See Marciel (n 25).

188 Eric Heinze, *Hate Speech and Democratic Citizenship* (OUP 2016) 46–7, cited in Kenyon (n 73) 67 fn 21.

189 Meiklejohn (n 183) 27, 75.

190
*Mouvement Raëlien Suisse v Switzerland* [2012] ECtHR [GC] 16354/06 (Dissenting Opinion of Judge Pinto de Albuquerque).

191
*Nit Srl v Republic of Moldova* [2022] ECtHR [GC] 28470/12 [175].

192
*Association Burestop* (n 146) para 85 [translation].

193 ibid (translation).

194
*Dink* (n 96) 137; *Uzeyir Jafarov v Azerbaijan* [2015] ECtHR 54204/08 68; *Khadija Ismayilova v Azerbaijan* [2019] ECtHR 65286/13, 57270/14 159; *Gaši and Others v Serbia* [2022] ECtHR 24738/19 78; Pentney (n 6).

195 Kenyon (n 73) 1.

196 World Economic Forum, ‘Global Risks Report 2024: 19th Edition Insight Report’ (2024) 18–21 <www.weforum.org/publications/global-risks-report-2024/in-full/global-risks-2024-at-a-turning-point/> accessed 10 January 2024; LSE Truth, Trust & Technology Commission, ‘Tackling the Information Crisis: A Policy Framework for Media System Resilience’ (The Report of the LSE Commission on Truth, Trust and Technology 2018) <lse.ac.uk/media-and-communications/assets/documents/research/T3-Report-Tackling-the-Information-Crisis-v6.pdf> accessed 15 December 2023.

